# The relation between Complex PTSD and Borderline Personality Disorder – a review of the literature

**DOI:** 10.1192/j.eurpsy.2022.1721

**Published:** 2022-09-01

**Authors:** B. Ramos, F. Santos Martins, A. Elias De Sousa, I. Soares Da Costa, F. Andrade

**Affiliations:** Centro Hospitalar Universitário São João, Psychiatry And Mental Health Department, Porto, Portugal

**Keywords:** borderline personality disorder, complex post-traumatic stress disorder, Post-traumatic stress disorder, Trauma

## Abstract

**Introduction:**

Adults diagnosed with Borderline Personality Disorder (BPD) likely have a history of psychological trauma. There has been research about the connection between Complex Post-Traumatic Stress Disorder (c-PTSD) and BPD.

**Objectives:**

This paper provides a review of the relationship between complex trauma and key features of BPD.

**Methods:**

Review of the literature from 2015 to present, using search engines such as Pubmed and Google Shoolar, using the following keywords: borderline personality disorder, complex post-traumatic stress disorder, trauma

**Results:**

Traumatic victimisation and compromised primary caregiving relationships have been hypothesized to be key aetiological factors in the subsequent development of BPD. c-PTSD was defined as a syndrome with symptoms of emotional dysregulation, dissociation somatisation and poor self-esteem, with distorted cognition about relationships, following traumatic interpersonal abuse. It was proposed as an alternative for understanding and treating people who had suffered prolonged and severe interpersonal trauma, many of whom were diagnosed with BPD. Although, the boundaries between c-PTSD and BPD remain vague. Currently, the main difference is the assumption that symptoms of c-PTSD are sequelae of exposure to traumatic stress, which is not inherent in the current DSM-5 definition of BPD. Furthermore, to date, the neurochemistry and neurostructural changes seen in c-PTSD, BPD and PTSD do not clearly differentiate the three conditions.

**Conclusions:**

BPD and PTSD are relatively distinct with regard to the precise qualitative definitions of their diagnostic features, but nevertheless have substantial potential overlap in their symptom criteria.

**Disclosure:**

No significant relationships.

